# Research Advances in Bionic Cell Membrane-Mediated Nanodrug Delivery Systems for the Treatment of Periodontitis with Osteoporosis

**DOI:** 10.3390/ijms27020583

**Published:** 2026-01-06

**Authors:** Xinyuan Ma, Dingxin Xue, Siqi Li, Guangxin Yuan, Yufeng Ma

**Affiliations:** 1 School of Stomatology, Shanxi Medical University, Taiyuan 030001, China; xinyuan_ma01@163.com; 2 College of Medical Imaging, Shanxi Medical University, Taiyuan 030001, China; xdx0907@163.com; 3 Basic Medical College, Beihua University, Jilin 132013, China; 18946720695@163.com; 4 College of Pharmacy, Beihua University, Jilin 132013, China

**Keywords:** bionic cell membrane-coated nanoparticles, targeted drug delivery, periodontitis-osteoporosis synergistic therapy, bone homeostasis regulation

## Abstract

With the intensification of global population aging, the co-morbidity rate of periodontitis and osteoporosis has significantly increased. The two are pathologically intertwined, forming a vicious cycle characterized by bone immunoregulatory dysfunction in the periodontal microenvironment, abnormal accumulation of reactive oxygen species (ROS), and disruption of bone homeostasis. Conventional mechanical debridement and anti-infective therapy can reduce the pathogen load, but in some patients, it remains challenging to achieve long-term stable control of inflammation and bone resorption. Furthermore, abnormal bone metabolism in the context of osteoporosis further weakens the osteogenic response during the repair phase, limiting the efficacy of these treatments. Bioinspired cell membrane-coated nanoparticles (CMNPs) have emerged as an innovative technological platform. By mimicking the biointerface properties of source cells—such as red blood cells, platelets, white blood cells, stem cells, and their exosomes—CMNPs enable targeted drug delivery, prolonged circulation within the body, and intelligent responses to pathological microenvironments. This review systematically explores how biomimetic design leverages the advantages of natural biological membranes to address challenges in therapeutic site enrichment and tissue penetration, in vivo circulation stability and effective exposure maintenance, and oxidative stress and immune microenvironment intervention, as well as functional regeneration supported by osteogenesis and angiogenesis. Additionally, we conducted an in-depth analysis of the key challenges encountered in translating preclinical research findings into clinical applications within this field, including issues such as the feasibility of large-scale production, batch-to-batch consistency, and long-term biosafety. This review lays a solid theoretical foundation for advancing the clinical translation of synergistic treatment strategies for periodontitis with osteoporosis and provides a clear research and development pathway.

## 1. Introduction

Periodontitis is a chronic inflammatory disease driven by bacterial plaque biofilm, which can cause destruction of periodontal supporting tissues and alveolar bone resorption, and is associated with various systemic metabolic and bone metabolic abnormalities [1,2,3]. Osteoporosis alters the host’s bone response pattern to inflammatory stimuli through imbalanced bone remodeling. Against a backdrop of estrogen deficiency, persistently elevated inflammatory mediators, and enhanced osteoclastic activity, alveolar bone becomes more susceptible to progressive resorption. The osteoblastic differentiation and mineralization processes during the repair phase are constrained, making it more difficult for periodontal regenerative therapy to achieve stable, reproducible histological structures [4]. Therefore, the pathogenesis of periodontitis with osteoporosis often arises not from a single factor, but rather from the combined effects of persistent bacterial load, prolonged inflammatory mediators, sustained oxidative stress, and bone metabolism biased toward resorption. Clinically, mechanical debridement and antimicrobial therapy can reduce pathogen load and alleviate symptoms to some extent. However, in some patients, unstable inflammation control and increased recurrence risk may still occur. Furthermore, for patients with concomitant osteoporosis, the bone resorption process under inflammatory conditions is more difficult to effectively curb [5,6]. This suggests that periodontal treatment must not only address pathogen eradication but also ensure more reliable drug delivery and retention at the lesion site, while providing quantifiable therapeutic effects on oxidative stress, immune responses, and bone remodeling processes.

Therefore, there is an urgent need for a novel therapeutic strategy capable of simultaneously modulating these complex pathological networks and providing a long-term effective treatment regimen. Nano-based drug delivery systems offer a novel technical approach to address the aforementioned challenges. Compared to conventional nanocarriers, cell membrane-coated nanocarrier delivery platforms establish a more defined structural foundation for circulatory stability, immunocompatibility, and tissue interactions by introducing the surface molecular profile of the donor cells at the nanoscale interface. This approach also provides scope for engineered designs tailored to the tumor microenvironment [7,8,9,10,11]. In periodontitis models, studies have reported that cell membrane coating systems can achieve combined antibacterial and anti-inflammatory effects, with efficacy supported by indicators such as inflammatory mediators and bone resorption [12,13]. Meanwhile, reactive hydrogels can link drug release to the oxidative stress levels at the lesion site, enabling more targeted release in high ROS environments [14,15]. Exosomes, as cell-free carriers for delivery and signaling, possess a characteristic lipid bilayer protection and engage in endocytic interactions with target cells. They have been systematically elucidated in studies related to bone regeneration and angiogenesis, giving rise to various engineered strategies, such as scaffold composites and targeted enhancement [16]. Additionally, plant-derived exosome-like vesicles have been employed in periodontitis models to alleviate inflammation and oxidative stress, primarily through improvements in antioxidant enzyme expression and tissue damage [17,18]. These strategies collectively reflect a trend where therapeutic design is progressively shifting from single-target approaches—such as antimicrobial or anti-inflammatory agents—toward comprehensive regulation of the lesion microenvironment and regenerative conditions.

This review systematically explores the latest research advances in biomimetic cell membrane delivery platforms for synergistic antibacterial, anti-inflammatory, antioxidant, and osteogenic promotion effects. Simultaneously, the existing bottlenecks in translating preclinical applications to clinical settings will be comprehensively addressed. This includes scalable acquisition of membrane-derived materials and process stability for coating techniques, defining quality attributes for batch consistency in formulations, and establishing and validating a long-term biosafety evaluation system.

## 2. Overview of Cell Membrane-Inspired Nanoparticles

Cell membrane-inspired nanoparticle technology offers new prospects for disease treatment by mimicking the biointerface characteristics of natural cells. The core principle of this technology involves completely encapsulating purified cell membranes (derived from red blood cells, platelets, white blood cells, macrophages, neutrophils, stem cells, etc.) onto the surface of synthetic nanoparticles, forming a core–shell structure with “bionic recognition–smart delivery” capabilities. This unique design endows the nanocarrier with dual advantages. On one hand, it inherits the source cells’ inherent targeting molecules and immunomodulatory functions, significantly enhancing drug accumulation efficiency at the lesion site. On the other hand, by leveraging the dynamic response characteristics of membrane structures, precise alignment between drug release and changes in the pathological microenvironment is achieved, providing an innovative tool for the coordinated intervention of complex pathological processes, such as tumors, inflammation, and bone metabolic diseases.

### Pharmacokinetics of Cell Membrane-Coated Nanoparticles

The construction of cell membrane-mimetic nanoparticles (CMNPs) requires precise regulation of interactions between the membrane and core interfaces to maintain the functional activity of membrane proteins and the integrity of membrane structures. The current overview of primary carrier selection and preparation strategies is summarized in Table 1 [19,20,21,22,23,24,25].

**Figure 1:**
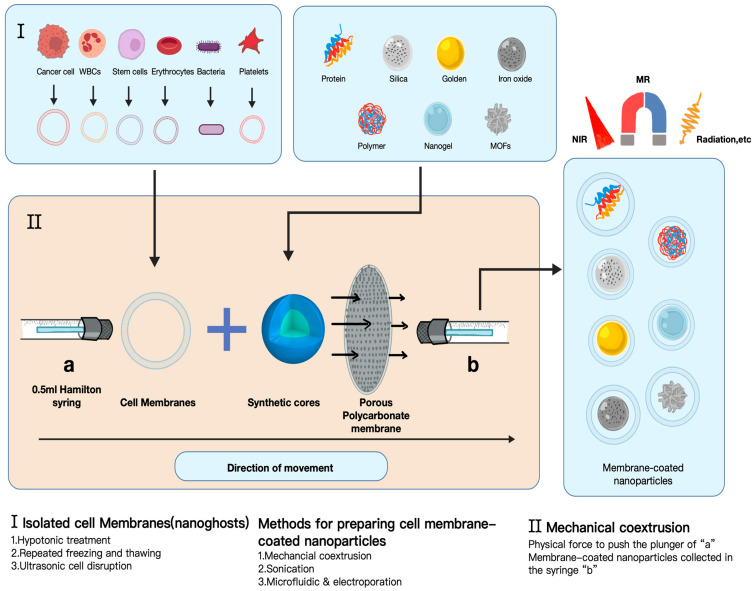
The physical co extrusion method for film coating preparation and the preparation process of biomimetic nanoparticles. The schematic was created using elements from BioRender.com and finalized in Procreate v.5.3.10. Created in BioRender. Li, P. (2026) https://BioRender.com/gb0y2sy (accessed on 25 November 2025).

Cell membrane-coated nanoparticle technology primarily relies on biomimetic self-assembly strategies, with membrane extrusion, ultrasound-assisted methods, and microfluidic electroporation representing three representative preparation techniques (Figure 1).

(1) The membrane extrusion method: Utilizing porous polycarbonate membranes for mechanical extrusion, fluid shear forces drive the self-assembly of cell membranes with nanocore materials to form uniform encapsulation structures. This method offers strong control over membrane orientation, but the limited throughput of the associated equipment restricts the possibility of large-scale production.
(2) The ultrasound-assisted method: Utilizes the cavitation effect to disrupt the bilayer structure of cell membranes, enabling their reassembly and encapsulation onto nanoparticle surfaces. Ultrasonic power and duration must be strictly controlled to prevent denaturation of membrane proteins or loss of drug activity.
(3) Microfluidic electroporation: By applying a pulsed electric field within microchannels, it transiently increases cell membrane permeability, facilitating the transmembrane transport of nanocore materials and their fusion with membrane components. This technology demonstrates excellent monodispersity and high encapsulation efficiency, making it particularly suitable for the precise construction of functional composite carriers.

To ensure the rationality and effectiveness of membrane-encapsulated nanoparticles in design and application, a systematic evaluation of their physicochemical properties and biological functions is required. Morphological analysis typically employs transmission electron microscopy (TEM) to visually verify the integrity of the core–shell structure and the uniformity of its encapsulation. Surface properties were characterized by zeta potential and dynamic light scattering (DLS), respectively, evaluating changes in surface charge before and after encapsulation, as well as hydrodynamic size and dispersion stability. For functional validation, nanoparticle tracking analysis (NTA) enables real-time monitoring of particle concentration and size distribution, while Western blotting quantifies the retention of membrane proteins to confirm their biological activity [23,24,25].

Currently, although nanomedicines have demonstrated promising efficacy in both in vitro and in vivo studies, their translation into clinical applications remains limited. A key reason is the lack of a comprehensive pharmacokinetic evaluation system capable of fully reflecting the in vivo dynamic behavior of bionic nanomedicines. Unlike traditional drugs that merely monitor total drug concentration, bionic nanodelivery systems undergo complex dynamic processes within the body, including storage in encapsulated forms, targeted release, and the transformation of their effect mechanisms. Therefore, establishing appropriate pharmacokinetic analysis methods is crucial. The use of high-performance liquid chromatography (HPLC) to quantify drug loading capacity, along with fluorescence resonance energy transfer (FRET) technology to study real-time drug release kinetics, holds significant importance for advancing the clinical translation of these technologies.

It is particularly important to emphasize that the construction of cell membrane-mimetic nanoparticles should adhere to the principle of “disease-oriented design”. Specifically, this indicates designs where the source of the cell membrane, the core material and drug delivery strategy are optimized in a target manner and based on the pathological characteristics of the target tissue, such as the pH of the inflammatory microenvironment and the expression profile of bone resorption-related enzymes. This customized design philosophy not only enhances the precision of treatment but also provides more effective solutions for clinical care.

## 3. Synergistic Therapeutic Strategies Targeting the Pathological Microenvironment of Periodontitis and Osteoporosis

The pathological processes of periodontitis and osteoporosis are intertwined, forming a vicious cycle. The chronic inflammation of periodontitis not only leads to alveolar bone loss but also accelerates the progression of systemic osteoporosis, while osteoporosis itself exacerbates the degenerative changes in periodontal tissues. This bidirectional interaction makes it difficult for a single treatment approach to break this vicious cycle. Current clinical treatment methods, such as mechanical debridement, antibiotic therapy, and tissue regeneration techniques, can alleviate symptoms to a certain extent. However, they often fail to address the root cause of the disease, due to issues like prolonged treatment cycles, short-lived effects, and high recurrence rates. To achieve more effective treatment, there is an urgent need to develop multi-targeted, synergistic therapeutic strategies capable of simultaneously regulating the complex pathological relationship between periodontitis and osteoporosis.

In recent years, bionic nanotechnology has provided a new therapeutic approach. Through the biomimetic cell membrane-coated nanoparticle (CMNP) platform, precise targeting of pathological sites can be achieved, modulating the immune microenvironment, alleviating oxidative stress, and synergistically promoting osteogenesis and angiogenesis. This enables the combined treatment of periodontitis and osteoporosis [26,27].

This review will elaborate in detail how these bionic nanodelivery platforms restore homeostasis of periodontal bone tissue through precise targeting, immune modulation, oxidative stress, and the synergistic effects of osteogenesis and angiogenesis, based on different pathological mechanisms.

### 3.1. Precisely Targeting Inflammation and Bone Injury Sites

Following systemic or local administration, drug distribution within periodontal pockets, inflammatory exudate tissues, and bone defect areas is frequently influenced by blood perfusion, tissue barriers, biofilm encapsulation, and nonspecific uptake. This results in insufficient effective concentrations at the target site or inadequate duration of action [7]. The value of biomimetic cell membrane carriers lies in their ability to retain the parent cell’s adhesion molecules and receptors on their surface. This enables selective interaction with inflammatory endothelial activation molecules and injury-related signals, thereby enhancing enrichment efficiency in the lesion area while reducing ineffective distribution [28].

#### 3.1.1. Bionic Nanoparticle Delivery System Mimicking Platelet Membranes

**Figure 2:**
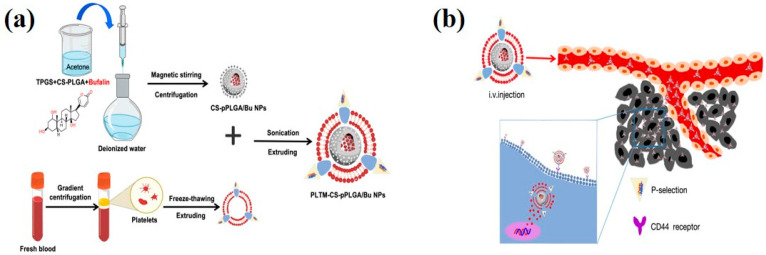
(**a**) Schematic illustration of the preparation route to PLTM-CS-pPLGA/Bu NPs; (**b**) in vivo targeted bufalin delivery to a tumor site mediated by binding of P-selectin on the surface of the PLTM to CD44 receptors of the tumor cells. The schematic was created using elements from BioRender.com and finalized in Procreate. Created in BioRender. Li, P. (2026) https://BioRender.com/gb0y2sy (accessed on 25 November 2025).

Platelets originate from the nucleated cell fragments of bone marrow megakaryocytes and experience targeted enrichment following vascular injury and inflammatory adhesion. Although they constitute only 0.5–1% of total blood cells in circulating blood, they play a crucial role in hemostasis, thrombosis, and vascular injury repair [29]. Research indicates that platelet membranes, due to their surface-specific receptors (such as CD41, CD61, P2Y12, and GPIIb/IIIa) conferring injury recognition capabilities, serve as ideal biological carriers for targeting the vascular system [30] (Figure 2a). Under inflammatory stimulation, platelets can release nanoscale-derived particles and retain their ability to adhere and target inflammatory sites [31]. Compared to erythrocyte membrane carriers, platelet membranes exhibit a relatively shorter circulation time but respond more rapidly to vascular injury and endothelial activation. Consequently, they are better suited for drug delivery scenarios requiring rapid localization, particularly demonstrating theoretical advantages in dual-pathology contexts such as periodontitis with osteoporosis, where both inflammatory responses and tissue damage coexist [32]. In a specific study, Wang et al. [33] constructed platelet-like membrane-mimetic nanoparticles to deliver bufadienol D. By expressing CD47 on the membrane surface, they reduced immune clearance and achieved targeted enrichment through P-selectin–CD44 interactions, thereby enhancing tissue accumulation and reducing toxicity (Figure 2b). Hu et al. [34] further indicated that platelet-derived coatings can reduce macrophage phagocytosis but may be associated with complement activation under certain conditions, suggesting that their in vivo immunocompatibility requires evaluation in conjunction with preparation processes and administration contexts. The platelet-rich fibrin material developed by Choukroun et al. [35] systematically demonstrated the potential of platelet-derived materials to promote tissue repair and healing, providing fundamental evidence for the application of platelet-related strategies in regulating the regenerative microenvironment [36].

In summary, the platelet membrane-associated system excels at addressing the challenge of “difficulties in localization caused by endothelial activation and injury adhesion”. Its core contribution lies in enhancing the efficiency of early lesion localization. However, its circulation time and immunocompatibility are influenced by material sources, membrane protein status, and the in vivo environment, necessitating complementary strategies for long-term maintenance.

#### 3.1.2. Leukocyte-Membrane-Inspired Nanoscale Delivery System: Active Enrichment Driven by Inflammatory Chemotaxis and Adhesion

Breakthroughs in cell membrane engineering have enabled researchers to construct functionalized nanocarriers using diverse membrane sources, including stem cells, immune effector cells, coagulation-associated platelets, and pathogenic microorganisms [37,38,39]. Among these, immune cell membranes retain cytokine receptors, adhesion molecules, and immune recognition-related molecules, offering advantages in enhancing enrichment within inflammatory regions and reducing non-specific clearance [40,41]. In 2013, Parodi et al. [42] first reported leukocyte-membrane-coated biomimetic nanoparticles. This system bypasses phagocyte conditioning, reduces clearance by the mononuclear phagocyte system, and avoids the lysosomal degradation pathway. Consequently, it enhances drug accumulation in inflammatory tissues and prolongs circulation time in vivo. The surface antigens on white blood cell membranes confer natural targeting properties to the delivery system. Combined with its inherent ability to neutralize inflammatory mediators, this significantly enhances anti-inflammatory and anti-infective effects. The most extensively studied leukocyte sources are primarily macrophages and neutrophils: the former can mimic phagocytic behavior to accumulate at pathological sites [43], while the latter can be recruited early to inflammatory or tumor sites driven by chemokines and adhesion molecules [44].

Macrophages, as one of the core cells of the innate immune system, possess the ability to traverse barriers and enter diseased tissues [45,46,47]. Building upon this foundation, the macrophage membrane-enveloping system is not only employed for localization but also for the regulation of inflammatory mediators. Rao et al. [48] constructed membrane-modified magnetic nanoparticles using gene-edited macrophages overexpressing SIRPα. By competitively binding to CD47 on tumor cell surfaces, these nanoparticles reversed immune evasion, demonstrating that membrane receptors can be harnessed to amplify specific immune interactions. The Yin team [49] encapsulated Resolvin D1 within gold nanocages and delivered them into the membranes of lipopolysaccharide-pretreated macrophages. Leveraging Toll-like receptor-mediated recognition on the membrane surface, the nanocages accumulated at inflammatory sites. By neutralizing pro-inflammatory factors and inducing M2 polarization, they improved the cytokine environment, thereby promoting bone repair and regeneration. Corresponding to the chemotactic axis associated with periodontitis, Wang et al. [50] reported that CCL2-clearance-type nano-bait hydrogels can regulate monocyte recruitment and influence macrophage polarization states, providing verifiable materials science evidence for inflammatory cell migration and phenotypic regulation in periodontitis.

Neutrophils are another type of white blood cell widely utilized in cell membrane nanotechnology. As the most abundant white blood cells in the circulatory system, they can rapidly migrate to the site of inflammation and participate in phagocytosis and cytokine release during inflammatory responses [51]. In recent years, targeted strategies based on the neutrophil membrane (NM) have been continuously expanded. Gao et al. [52] employed NM coating to construct biomimetic nanovesicles, loading Resolvin D1 onto the membrane surface and cefotiam within the vesicles to achieve precise targeting of inflammatory sites. This approach significantly enhanced local bacterial clearance and inflammation resolution. The Ju team [53] reported that NM-derived nanovesicles can achieve targeted tissue damage repair, demonstrating their potential for clinical translation. Zhou et al. [54] integrated the drug-loading advantages of tetrahedral framework nucleic acids with NM properties, significantly enhancing the bioavailability of baicalin while maintaining effective concentrations and promoting the phenotypic shift from pro-inflammatory M1 to anti-inflammatory M2 macrophages. Further research by Gao et al. [55] utilized an NM nanovesicle delivery system in a vascular inflammation model. By activating the NF-κB pathway in endothelial cells to upregulate ICAM-1, the system achieved active targeting through binding to integrin β2 on the NM surface, thereby alleviating the inflammatory response. The above studies collectively demonstrate that the NM system is better suited for responding to inflammatory chemotaxis and adhesion signals, enabling early and rapid enrichment at inflammatory sites. Notably, the targeting capability of the neutrophil membrane system depends on the conformation and functional integrity of key membrane proteins; protein denaturation during preparation may directly reduce targeting efficiency and introduce immunogenic risks.

In summary, the bionic nanodelivery systems mimicking platelet and leukocyte membranes are primarily designed to address insufficient drug delivery caused by the complex anatomical structure and multiple tissue barriers in the periodontal region. This is based on the retention of adhesion molecules and inflammation-related recognition receptors on the membrane surface, enabling the carrier to adhere and reside more readily within inflammation-activated vascular endothelium, extravasation areas, and tissues surrounding bone defects. This enhances effective delivery within the lesion site while reducing distribution to non-target areas. Macrophage membranes are better suited for balancing localization and inflammatory mediator regulation, while neutrophil membranes are more appropriate for rapid enrichment during the early stages of inflammation. Both types are highly sensitive to the functional integrity of membrane proteins, and the quality of their preparation and characterization directly determines their reproducibility and safety.

### 3.2. Prolonged Systemic Circulation and Enhanced Focal Accumulation

Even when equipped with targeting ligands or adhesion capabilities, nanoscale systems may still be rapidly recognized and cleared by the mononuclear phagocytic system (MPS) in vivo, resulting in insufficient effective exposure and difficulty in sustaining therapeutic efficacy at the target site. Red blood cell membrane-mimetic systems, owing to their inherent long circulation characteristics, have been employed to mitigate this type of exposure loss.

#### Red Blood Cell Membrane-Inspired Nanoscale Delivery System: Delivery Enhancement Driven by Long Circulation and Immune Evasion

Red blood cells are the most abundant cellular component in blood, with a lifespan of approximately 100 to 120 days. They possess excellent biocompatibility and high loading capacity potential, making them a natural delivery interface [56]. As one of the earliest developed bionic nanocarriers, RBCM effectively suppresses MPS recognition and clearance through specific interaction between surface CD47 and macrophage signaling regulator protein α (SIRPα), thereby significantly enhancing the in vivo circulation stability of nanoparticles [57,58].

In periodontal infection delivery, Tang et al. [59] developed a gingipan-targeted liposome system encapsulated with red blood cell membranes to inhibit *Porphyromonas gingivalis*. RBC@GLR nanovesicles disrupt bacterial iron metabolism via porphyrin gallium and induce ROS production upon blue light activation, achieving synergistic bactericidal effects while reducing off-target cytotoxicity and minimizing resistance risks. The study also leveraged the biological characteristics of *Porphyromonas gingivalis* regarding red blood cell adhesion and hemoglobin utilization to achieve more targeted pathogen delivery. Previous studies have demonstrated that Fang et al. [60] incorporated folic acid into red blood cell membranes to construct RBC-NPs targeting oral tumor cells, indicating that red blood cell membranes possess a certain degree of surface engineering potential. Professor Zhang’s team [61] developed a nano-sponge centered on PLGA and coated with red blood cell membranes. In vivo experiments demonstrated its ability to eliminate short-term inflammatory responses and protect host cells by adsorbing and neutralizing bacterial toxins and inflammatory mediators, revealing its potential value in reducing material-related inflammatory reactions.

In bone metabolism-related delivery, Pan et al. [62] employed red blood cell membrane-coated PLGA nanoparticles to enhance in vivo circulation stability and reduce phagocytosis by immune cells. The mechanism involves this nanosystem inhibiting NF-κB- and MAPK-related phosphorylation processes while enhancing the osteogenic differentiation capacity of hPDLSCs, thereby promoting BMSC-mediated bone regeneration in bone defect models. This study provides mechanistic support for the role of red blood cell membrane-coated systems in osteoporosis-related repair, extending beyond purely pharmacokinetic investigations [41,63,64].

Overall, red blood cells possess long-term circulation and an “autologous labeling” immune exemption characteristic, addressing issues such as short drug retention time in vivo and rapid clearance that lead to the loss of an effective dosage. This provides a natural biological interface for targeted drug delivery, inflammatory factor neutralization, and tissue regeneration. Additionally, RBCM can reduce material-related inflammatory responses in complex periodontal lesions by adsorbing and neutralizing toxins or inflammatory mediators, thereby achieving more stable local effects.

### 3.3. Alleviating Oxidative Stress and Remodeling the Immune Microenvironment

The microenvironment of periodontitis lesions with osteoporosis often exhibits a superimposed state of oxidative stress and chronic inflammation. Excessive ROS not only directly damages cellular structures and disrupts mitochondrial homeostasis, but also amplifies inflammatory signals and activates osteoclast-related processes; this persistent inflammatory cascade further promotes ROS production and exacerbates local tissue damage [65,66,67]. Given their mutually reinforcing relationship, relying solely on antimicrobial or anti-inflammatory strategies often proves insufficient to stably reverse microenvironmental imbalances. Therefore, delivery and material designs targeting this module should focus on reducing ROS burden, attenuating pro-inflammatory stimuli, and redirecting immune responses toward repair, thereby creating more sustainable local conditions for subsequent bone regeneration.

#### 3.3.1. Macrophage Membrane Nanoparticle Delivery Platform: From Inflammatory Factor Neutralization to Immune Phenotype Remodeling

Macrophage membranes possess a complex network of surface receptors, endowing them with a natural affinity for sites of inflammation and reducing the immunogenicity of nanocarriers [68,69]. Building upon the aforementioned inflammatory homing mechanism, the macrophage membrane platform is further utilized for “microenvironmental reprogramming”. For example, the Yin team utilized lipopolysaccharide-pretreated macrophage membranes to encapsulate Resolvin D1 within gold nanocages. This approach enhanced enrichment in inflammatory regions through Toll-like receptor-mediated recognition on the membrane surface, neutralized pro-inflammatory factors, and induced reparative polarization. Consequently, it improved the cytokine environment and promoted bone repair. Focusing on chemotaxis axis intervention, Wang et al. developed CCL2-clearance nano-bait hydrogels capable of influencing monocyte recruitment and regulating macrophage polarization, providing materials science evidence for inflammatory cell migration and phenotypic regulation in periodontitis. Encapsulated naringenin-loaded hollow mesoporous polydopamine nanoparticles within macrophage membranes to construct a composite system. This approach retained the ROS scavenging capacity of the nanoparticle core while inheriting the inflammation-related enrichment properties of the macrophage membrane. Consequently, the composite demonstrated synergistic effects in reducing ROS levels, protecting surrounding tissues, and suppressing secondary infections. Wang et al. [70] found that exosomes secreted by macrophages stimulated by titanium dioxide nanotubes enhanced alkaline phosphatase activity in BMSCs and upregulated osteogenesis-related genes, suggesting that immune cell-associated secretions may play a direct role in regulating bone regeneration signaling. Lin et al. [71,72] achieved bactericidal effects, alleviated hypoxia, and eliminated excess reactive oxygen species (ROS) by modifying the antimicrobial peptide LL-37 on the macrophage membrane surface and encapsulating L-amino acid oxidase with hollow manganese dioxide to form a core–shell nanobait system, thereby promoting periodontal tissue regeneration.

Overall, the macrophage membrane nanoplatform addresses the microenvironment of periodontitis-associated osteoporosis. Its advantage lies in simultaneously regulating inflammatory mediators, reducing ROS levels, and maintaining the dynamic equilibrium between pro-inflammatory and pro-repair phenotypes. This provides an actionable materials science approach for “bone-immune intervention” in periodontitis-related osteoporosis.

#### 3.3.2. Stem Cell Membrane and Inorganic Nanocore: Coupling of ROS Scavenging and Inflammatory Factor Neutralization

Stem cells are widely utilized in regenerative medicine research due to their multipotent differentiation potential, migratory homing capacity, and paracrine regulatory functions [73,74]. In the pathological context of periodontitis with osteoporosis, elevated pro-inflammatory cytokines frequently coexist with reactive oxygen species accumulation. Together, they impair cellular function and exacerbate tissue destruction. Therefore, intervention strategies must simultaneously reduce oxidative stress levels and mitigate inflammation-mediated persistent stimulation. Li et al. [27] constructed a MnO_2_@hPM functionalized nanoplatform, utilizing periodontal ligament stem cell membranes as the outer interface and incorporating MnO_2_ as the inorganic core. This system exhibits targeted enrichment capabilities at periodontal inflammation sites, neutralizing pro-inflammatory factors and scavenging excess ROS. This improves mitochondrial function and enhances the osteogenic differentiation capacity of periodontal ligament stem cells, ultimately boosting tissue regeneration efficacy. Compared to approaches relying solely on anti-inflammatory or antioxidant mechanisms, this design better aligns with the pathological characteristics of concurrent inflammatory responses and oxidative damage.

#### 3.3.3. Hydrogel Microenvironment Engineering: ROS-Responsive Release and Immune Phenotype Guidance

**Figure 3:**
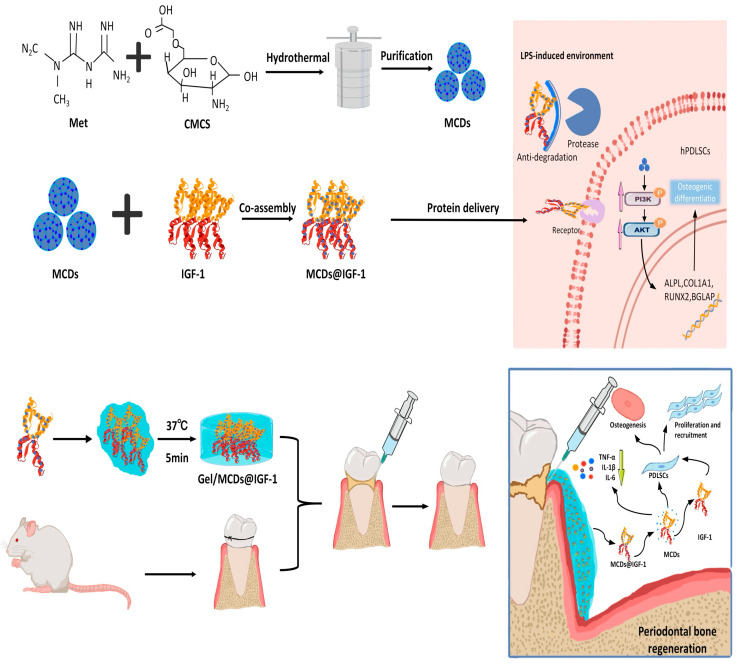
Schematic illustration of the Gel/MCDs@IGF-1 composite hydrogel system and its dual-action mechanism for enhanced periodontal bone regeneration under inflammatory conditions. The schematic was created using elements from BioRender.com and finalized in Procreate. Cre-ated in BioRender. Li, P. (2026) https://BioRender.com/gb0y2sy (accessed on 25 November 2025).

Hydrogels possess excellent pore structures, biocompatibility, and controllable release characteristics, making them suitable as delivery carriers for active agents such as growth factors, cells, and exosomes, and enabling their use in regulating local regenerative microenvironments [75] (Figure 3). Its mechanism involves directly scavenging excess ROS to alleviate oxidative stress and protect stem cells, while also reprogramming pro-inflammatory M1 macrophages into anti-inflammatory, reparative M2 macrophages, thereby reshaping the microenvironment. This synergistic effect creates an optimal state characterized by low oxidative stress, anti-inflammatory properties, and abundant growth factors, ultimately promoting angiogenesis and osteogenic differentiation of stem cells to achieve highly efficient bone defect repair. Abnormally elevated ROS levels in periodontal disease lesions can perpetuate inflammation and interfere with the repair process. Therefore, a ROS-triggered local delivery system can achieve more targeted drug release within the affected area. Zhu et al. [14] reported a ROS-responsive injectable hydrogel that forms a borate ester crosslinking structure, using 3-aminophenylboronic acid and polyvinyl alcohol, and co-loads minocycline hydrochloride with quercetin iron nanoparticles. This system can reduce ROS levels and promote reparative polarization by regulating Nrf2 and NF-κB-related signaling pathways, while simultaneously enhancing the osteogenic differentiation of hPDLSCs. Animal studies have demonstrated that it can inhibit alveolar bone resorption and promote the expression of osteogenic factors. Beyond single-drug delivery systems, the combination of hydrogels with extracellular vesicles also offers a viable approach for repairing periodontal defects. Shen et al. [76] developed a composite of dental pulp stem-cell-derived exosomes and chitosan hydrogel to enhance immunomodulatory effects through sustained local release. This approach reduced pro-inflammatory factor levels and inhibited osteoclast-related processes, thereby improving the efficiency of alveolar bone defect repair. In the field of anti-infective research, Yan’s team [77] treated mesenchymal stem cells with *Porphyromonas gingivalis* lipopolysaccharide and encapsulated metronidazole to construct a targeted nanogel. Leveraging pathogen-associated molecular pattern receptors, this approach enhances local antibacterial efficacy and biofilm clearance efficiency. The sustained-release structure prolongs local retention and improves pharmacokinetic performance.

Overall, hydrogel delivery platforms can address issues of fluctuating effective concentrations and irritation levels within inflammatory microenvironments. They enhance localized effective exposure and enable temporally and spatially controlled release, thereby suppressing inflammation and oxidative stress while providing a more favorable spatiotemporal order for subsequent angiogenesis and osteogenesis processes.

#### 3.3.4. Plant-Derived Exosome-like Nanovesicles: Low-Immunogenicity Carriers with Natural Antioxidant–Anti-Inflammatory Activity

In recent years, in addition to inorganic nanoenzymes and biomimetic cell membrane delivery systems, plant-derived exosome-like nanoparticles (ELNs) have increasingly been employed for interventions targeting the periodontitis-associated microenvironment [78,79]. These vesicles can be isolated from plant tissues or sap, exhibiting nanoscale membrane structures and displaying certain similarities in delivery behavior to mammalian extracellular vesicles. Its broad sources and relatively low immunogenicity make it feasible for application in localized oral diseases. Yu et al. [80] reported that garlic-derived ELNs can modulate PI3K/AKT-related pathways and produce anti-inflammatory and antioxidant effects, while improving metabolic-related phenotypes. Xie et al. demonstrated that ginger-derived exosome-like nanoparticles can upregulate the expression of antioxidant enzymes such as SOD-1 and CAT in periodontal ligament fibroblasts, thereby reducing ROS levels and enhancing cell migration capacity.

The common goal of nano-delivery systems utilizing different materials is to mitigate the adverse effects of the microenvironment created by the mutual promotion of persistent inflammation and oxidative stress in periodontitis complicated by osteoporosis. This approach aims to reduce the dominance of bone resorption and restore the function of osteoblast-related cells. Macrophage membrane-associated systems can reduce inflammation by binding or neutralizing pro-inflammatory mediators and inducing macrophages to transition toward repair-related phenotypes. Inorganic components such as MnO_2_ can mitigate excessive local reactive oxygen species and improve hypoxia-related limitations. ROS-responsive hydrogels can immobilize antimicrobial, antioxidant, and anti-inflammatory interventions at the lesion site for sustained release, enhancing local effective concentrations. Plant-derived exosome-like nanovesicles serve as a supplementary pathway with endogenous antioxidant and anti-inflammatory activity and low immunogenicity.

### 3.4. Synergistically Promoting Osteogenesis and Angiogenesis for Functional Regeneration

Following the resolution of inflammatory responses and oxidative stress, the structural repair of periodontal supporting tissues remains dependent on the restoration of blood supply and the process of bone formation. Insufficient vascular networks limit local oxygen and nutrient delivery, thereby impairing osteoblast function and matrix mineralization. Promoting angiogenesis alone without osteogenesis-related signaling support also hinders the formation of stable new bone structures. Therefore, delivery strategies during the regenerative phase often require simultaneous consideration of the sustained presence and release timing of both proangiogenic and pro-osteogenic signals within the defect area to support functional recovery of the alveolar bone and periodontal ligament complex.

#### 3.4.1. Exosome-Based Nanodelivery Systems: Integration of Cell-Free Regenerative Signaling with Tissue Homing

With the advancement of cell-free therapies, stem cell-derived exosomes are recognized as possessing regenerative potential comparable to progenitor cells [81]. Exosomes are nanoscale membrane vesicles secreted by living cells, typically measuring 30 to 150 nm in diameter, and constitute a major subclass of extracellular vesicles [82]. Their lipid bilayer membrane protects functional molecules such as microRNA, mRNA, and proteins, enabling them to exchange information with target cells through mechanisms like endocytosis, thereby influencing various repair-related processes. The surface molecular composition of exosomes correlates with their source cells. This variation influences their in vivo distribution and retention characteristics to some extent and may reduce the likelihood of rapid immune clearance [83]. Research indicates that exosomes can deliver therapeutic molecules more precisely to pathological sites while reducing systemic exposure risks [84]. In specific scenarios, engineered exosomes can also overcome certain physiological barriers, expanding the application boundaries of targeted delivery. In bone repair research, BMSC-derived exosomes can carry functional microRNAs and proteins to promote bone regeneration. Liu et al. [85] embedded BMSC-derived bone-inductive exosomes into mesoporous bioactive glass scaffolds for sustained release, enhancing bone regeneration outcomes. Their further analysis indicated that microRNAs such as let-7a-5p and let-7c-5p are associated with Smad1/5/9-related processes. Wang et al. [70] reported that combining MSC exosomes with hydrogel scaffolds significantly enhances bone defect repair. The Ge team [86] observed that hsa-mir-2110 and hsa-mir-328-3p levels increased with differentiation time in osteoblast-induced human umbilical cord MSC exosomes in an estrogen-deficient osteoporosis model, which was accompanied by activation of bone differentiation-related genes. In terms of targeted enhancement, Huang et al. [87] engineered CCR6-overexpressing gingival mesenchymal stem cell exosomes and fused them with grapefruit-derived exosome nanoparticles loaded with CX5461 to improve delivery efficiency. Considering the potential accumulation of stem-cell-derived exosomes in the liver and lungs in vivo, LUO et al. [88] conjugated BMSC-specific aptamers to exosomes to enhance their enrichment at injury sites and promote bone formation.

#### 3.4.2. Hydrogels and Composite Scaffolds: Sequential Release and Coordination of Angiogenesis and Osteogenesis Processes

**Figure 4:**
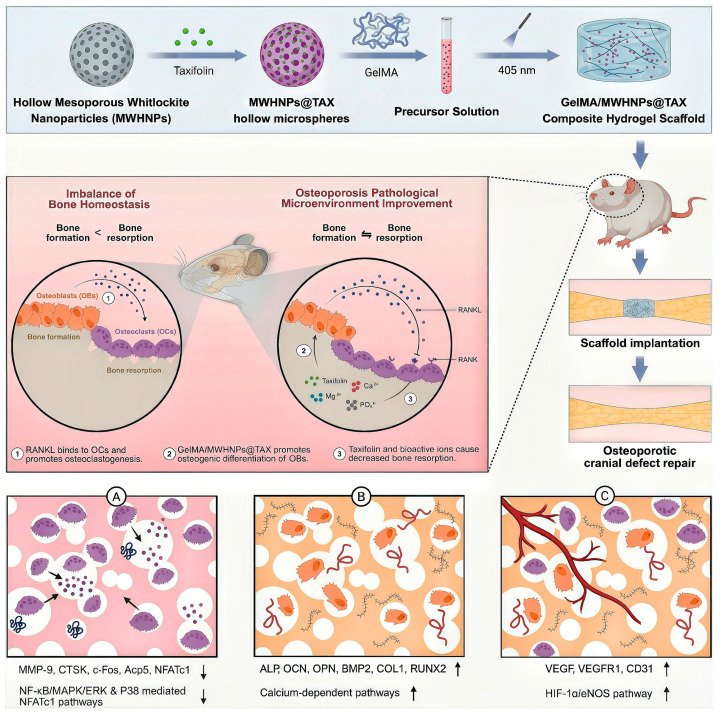
Schematic illustration of the fabrication of GelMA/MWHNPs@TAX composite hydrogels and their synergistic effects on osteoclastogenesis, osteogenesis and angiogenesis for osteoporotic cranial defect repair. (**A**) Inhibition of osteoclastogenesis and bone resorption. (**B**) Promotion of osteogenic differentiation and bone formation. (**C**) Enhancement of angiogenesis. Blue dots indicate osteoclastogenic signaling molecules (e.g., RANKL); ↑/↓ indicate increased/decreased expression or activity compared with the osteoporotic/control condition. Reprinted from Ref. [89] with permission.

Hydrogels can be utilized not only in the aforementioned microenvironment regulation phase but also in the regeneration phase as injectable fillers and controlled-release carriers, providing structural support to defect areas while enabling staged release of bioactive factors. Jiang et al. [89] (Figure 4) constructed a composite hydrogel by encapsulating mesoporous calcium hydroxide nanoparticles loaded with paclitaxel within methacrylate-modified gelatin. This system releases active molecules and bioactive ions, inhibiting osteoclast differentiation while promoting osteogenic differentiation of bone marrow mesenchymal stem cells (BMSCs) and enhancing endothelial cell migration and angiogenesis, thereby improving bone metabolic status.

Han et al. [90] developed an injectable composite hydrogel capable of sequential release of VEGF and pulp stem cell exosomes, which promoted angiogenesis and induced new bone formation in defect models while maintaining exosomal bioactivity throughout the release process. Placental mesenchymal stem-cell-derived exosomes combined with hydrogels also expand the scope of alveolar bone repair and increase the number and thickness of new trabeculae in periodontitis models [91]. Liu et al. [92] further demonstrated that gelatin-loaded BMSC extracellular vesicles promote periodontal tissue regeneration by modulating osteoclast function, macrophage polarization, and inflammatory immune responses. Another study combining 3D-printed hydrogels with black phosphorus, tricalcium phosphate, and exosomes demonstrated that tricalcium phosphate incorporation enhances scaffold elastic modulus while simultaneously promoting both osteogenic and chondrogenic differentiation of BMSCs within a dual-layer scaffold structure [93].

#### 3.4.3. Platelet-Derived Regenerative Materials: Mechanical Support and Early Angiogenesis Facilitation

Platelet-derived materials can provide mechanical support in defect areas and promote early angiogenesis. Chen Xingguang’s team [94,95] constructed β-GP@PM hydrogels to improve osteoporosis-related bone metabolic imbalance by regulating BMSCs’ chondrogenic differentiation and designed injectable GNP@PM hydrogels to achieve targeted drug delivery to bone defect sites. Leveraging platelet-rich fibrin membranes for structural support and promoting early angiogenesis, this approach accelerates bone regeneration.

#### 3.4.4. Stem Cells and Their Derived Signals: Dual Regulatory Functions in Osteogenesis and Vascularization

Recent studies have demonstrated that stem cell strategies not only promote ROS scavenging and inflammatory factor neutralization but also enhance bone regeneration through both direct differentiation and paracrine signaling. Research by Wei et al. [96] revealed that human periodontal ligament stem cells (hPDLSCs) not only possess osteogenic differentiation potential within inflammatory microenvironments but also serve as endogenous carriers to effectively deliver key signaling molecules such as insulin-like growth factor-1 (IGF-1). This synergistically promotes stem cell homing and localized bone formation. From a molecular mechanism perspective, exosomes derived from bone marrow mesenchymal stem cells (BMSCs) can efficiently activate the classical osteogenic pathway in target cells after loading with bone morphogenetic protein-2 (BMP-2) [97]. Animal studies further validate this approach: Lemaître et al. [98] successfully regenerated structurally intact periodontal tissue by co-implanting adipose-derived stem cells (ADSCs) with collagen scaffolds into defect sites, accompanied by significant upregulation of core osteogenic markers. For larger bone defects presenting greater clinical challenges, Kawaguchi et al. [99] employed a composite graft of autologous BMSCs and type I collagen, similarly achieving functional reconstruction of periodontal composite tissue.

However, direct stem cell transplantation faces clinical translation barriers such as stringent preservation conditions, potential tumorigenic risks, and complex regulatory requirements [100]. These limitations have driven research toward cell-free strategies. Derived vesicles such as stem-cell-derived exosomes can deliver functional nucleic acids, proteins, and lipids, enhancing formulation stability and targeting while preserving therapeutic efficacy. This offers a more promising translational solution for bone regeneration.

## 4. Discussion

The treatment challenges associated with periodontitis complicated by osteoporosis stem not from a single factor, but rather from the combined effects of persistent bacterial biofilm stimulation, prolonged maintenance of inflammatory mediators, elevated oxidative stress, and bone loss resulting from bone remodeling [2]. Against this backdrop, conventional mechanical debridement and anti-infective therapy can reduce the pathogen load, yet in some patients, it remains challenging to achieve stable control over local inflammation levels and bone resorption processes, resulting in poor treatment outcomes or high recurrence risks [101]. Osteoporosis-related bone metabolic imbalance exacerbates inflammatory bone resorption reactions and impairs osteoblast differentiation and matrix mineralization during the repair phase. Consequently, achieving stable and reparable histological structures in complex defect topographies becomes more challenging for periodontal regeneration [102].

In recent years, nanomedicines have achieved significant breakthroughs in the field of targeted therapy [7,103]. Cell membrane-coated nanoparticles essentially mimic the surface characteristics of the source cells, exhibiting excellent biocompatibility, homotypic targeting recognition, prolonged blood circulation, and immune evasion. This approach introduces the surface molecular profile of the source cells through the membrane interface, enabling the nanoparticle carriers to behave more closely to the authentic recognition and interaction patterns observed in biological organisms [104,105]. Compared to traditional synthetic nanocarriers, cell membrane-coated systems provide a clearer structural basis and greater engineering potential for enhancing circulation stability, immunocompatibility, and targeted tissue enrichment. In periodontitis-related research, studies have employed genetically engineered cell membrane-coated nanoparticles to simultaneously assess bacterial load/inflammatory factor levels and alveolar bone histology in filament ligature-induced periodontitis models. This suggests such platforms can achieve combined antibacterial and anti-inflammatory effects within the same model [13].

First, lesion enrichment determines the upper limit of effective local exposure. The reason platelet membranes and leukocyte membrane systems achieve higher distribution in inflammation- and injury-related tissues is due to the mutual recognition between adhesion molecules on the membrane surface and receptors on inflamed endothelial cells. For example, P-selectin binds to CD44, leading to enrichment in relevant pathological areas [106]. Neutrophil membranes upregulate the integrin molecule ICAM-1 through activation of the endothelial NF-κB signaling pathway, binding to it to facilitate adhesion during inflammatory processes and transmigration [52]. Macrophage membranes participate in the binding of endotoxins and pro-inflammatory mediators via interactions between membrane surface receptors and ligands, thereby regulating the effective concentration of inflammatory mediators during delivery [107].

Secondly, maintaining effective exposure within the body directly impacts drug accumulation at the target site and therapeutic stability. Early scientists first validated the feasibility of cell membrane encapsulation technology using erythrocyte membranes [108]. This characteristic does not “directly target” periodontal lesions, but it provides a non-toxic, specifically targeted, anti-inflammatory, and bactericidal effect requiring prolonged exposure time. Its anti-inflammatory mechanism primarily involves inhibiting the caspase-1-nuclear factor κB (Caspase-1-NF-κB) pathway, reducing oxidative stress, and alleviating inflammatory endothelial damage [109]. Persistent inflammation and immune microenvironment imbalance are key factors contributing to treatment failure. Existing research suggests that ROS load within lesions and the maintenance of inflammatory signaling exhibit a mutually reinforcing relationship. Single antimicrobial or anti-inflammatory approaches often fail to address the sources of tissue damage within this coupled context. Pathology-guided cell membrane-encapsulated nanoscale systems have been employed for comprehensive intervention in periodontitis, demonstrating enhanced inhibitory capacity against inflammation and tissue destruction within multi-synergistic strategies. Furthermore, nanosystems with reactive ROS scavenging capabilities have demonstrated the potential for immunomodulation in periodontitis models by regulating macrophage polarization [50,110,111]. ROS-responsive hydrogels establish a correlation between drug release and reactive oxygen species (ROS) levels, enabling enhanced drug release in high-ROS environments at the lesion site. This process is accompanied at the molecular level by Nrf2 upregulation and decreased NF-κB-related activation, ultimately manifesting as enhanced osteogenic differentiation of human periodontal ligament-derived stem cells (hPDLSCs) and inhibition of alveolar bone resorption [14]. These findings collectively demonstrate that the evaluation of microenvironmental interventions should not be confined to conceptual descriptions of anti-inflammatory or antioxidant effects. Instead, it should be grounded in evidence from quantifiable endpoints such as inflammatory mediators, ROS-related indicators, and markers associated with osteoclastic and osteoblastic processes.

The remarkable success of cell membrane-coated nanoparticles in applied research has propelled the advancement of drug delivery technology using stem cell exosomes. Scientists have ingeniously harnessed their inherent communication systems to load specific drugs, such as anticancer agents and nucleic acid therapeutics, via genetic engineering or chemical methods, enabling precise and efficient drug delivery to target sites [112,113].

When translating preclinical research into clinical practice, the feasibility of such bionic nanoengineering is not solely determined by specific therapeutic efficacy. To ensure the rationality and effectiveness of membrane-encapsulated nanoparticles in design and application, a systematic evaluation of their physicochemical properties and biological functions is required. It also hinges on issues such as large-scale production, batch consistency, and long-term biological safety. To ensure the rationality and effectiveness of membrane-encapsulated nanoparticles in design and application, a systematic evaluation of their physicochemical properties and biological functions is required [114]. Regarding immunogenicity, factors such as complement activation, hemolysis, endotoxin control, and distribution and clearance in major organs must be considered to evaluate the risk of immune responses and organ accumulation under repeated dosing conditions. Overall, biomimetic nanoparticles based on cell membranes have demonstrated broad application prospects in disease diagnosis, treatment, and prevention, exhibiting strong practicality and clinical translational value.

## 5. Conclusions

The research on CMNPs is still in the preclinical stage, and the translational clinical application is still in the exploratory stage. In the future, key issues such as the development of large-scale cell membrane production technology, the improvement of carrier formulation stability, and the verification of delivery efficiency in complex physiological environments need to be emphasized in order to promote the clinical treatment of similar diseases.

## Figures and Tables

**Table 1:** Application Studies of Cell Membrane-Coated Nanocarriers.

Carrier	Advantages	Disadvantages
PLGA	Enhancing drug efficacy, achieving sustained release, promoting biodegradability, reducing drug dosage, and minimizing adverse reactions	Processing temperature sensitivity
AuNC	Outstanding catalytic performance, excellent biocompatibility, significant potential for environmental applications, and exceptional optical properties	Relatively high cost and poor stability
PD	Environmentally sound and highly water-resistant	High cost
MnO_2_	Excellent biocompatibility, high targeting efficiency, biodegradable	Limited drug loading capacity, complex release mechanism
tFNA	High-efficiency delivery, excellent biocompatibility, structural stability	Clinical application remains at the trial stage.
Exosomes	High stability, strong hydrophilicity, potent targeting capability, and extremely low immunogenicity	Relatively low levels of exosomes
Chitosan	Biocompatible, biodegradable, non-toxic	Solubility limitations, limited drug loading capacity

## Data Availability

The original contributions presented in this study are included in the article. Further inquiries can be directed to the corresponding authors.

## Abbreviations

The following abbreviations are used in this manuscript:

CMNPs	Cell membrane-coated nanoparticles
RBCM	Red Blood Cell Membrane
PM	Platelet membrane
LM	Leukocyte membrane
TEM	Transmission Electron Microscopy
DLS	Dynamic Light Scattering
NTA	Nanoparticle Tracking Analysis
HPLC	High Performance Liquid Chromatography
FRET	Fluorescence Resonance Energy Transfer
RBC-NP	Red blood cell membrane-derived nanosystem
SIRPα	Signal-Regulatory Protein Alpha
RBC@GLR	Red Blood Cell Membrane-coated Gingipain-targeted Liposomal System
*P. gingivalis*	*Porphyromonas gingivalis*
GaPP	Gallium Porphyrin
ROS	Reactive Oxygen Species
GLR	Erythrocyte-mimicking nanovesicles(Erythrocyte membrane-camouflaged gallium porphyrin-loaded liposomes)
PLGA	Poly(lactic-co-glycolic acid)
OC	osteoclast
NF-KB	nuclear factor -kappa B pathway
PMPs	Nanoscale Platelet-Derived Microparticles
PLTM	Platelet membrane-biomimetic nanoparticles
iPRF	Platelet-Rich Fibrin
LM-NPs	leukocyte membrane-coated nanoparticles
MPS	Mononuclear Phagocyte System
MM-NPs	Macrophage Membrane-Coated Nanoparticles
BANC@MM	Bioinspired Anti-inflammatory Nanocapsules
LPS	Pre-phase lipopolysaccharide
AuNC	Gold nanocages
M-CNP	Caffeic acid phenethyl ester-loaded nanoparticles
MM	Macrophage Membrane
NAR	Naringenin
hPDA NPs	Hollow Mesoporous Polydopamine Nanoparticles
BMSCs	Bone Marrow Mesenchymal Stem Cells
rhLL-37	Humanized Recombinant Antimicrobial Peptide LL-37
LAAO	L-Amino Acid Oxidase
hMnO_2_	Hollow Manganese Dioxide
NDs	Nanoscale Decoy System
RvD1	Resolvin D1
CEF	Ceftazidime
NM-NVs	neutrophil membrane-derived nanovesicles
tFNA	Tetrahedral Framework Nucleic Acid
APT	Ac-PGP-tFNA
ICAM-1	Intercellular Adhesion Molecule 1
NM-NPs	Neutrophil Membrane-Coated Nanoparticles
hPDLSCs	Human periodontal ligament stem cells
MCDs	Metformin Carbon Dots
IGF-1	Insulin-like Growth Factor-1
PDLSCm	Periodontal Ligament Stem Cell Membrane
BMP-2	Bone Morphogenetic Protein-2
ADSCs	Adipose-Derived Stem Cells
OPN	Osteopontin
EXOs	Exosomes
BMSC-OI-Exo	Osteogenic-Induced Exosomes Derived from Bone Marrow Mesenchymal Stem Cells
MBG	Mesoporous Bioactive Glass
GaELNs	Garlic-Derived Exosome-Like Nanovesicles
GELNs	Ginger-Derived Exosome-Like Nanoparticles
GMSCs-Exo	Gingiva-derived mesenchymal stem cell exosomes
BMSCs-Exos-apt	Conjugation of Bone Marrow Mesenchymal Stem Cell-Specific Aptamer with Exosomes
PBA	Phenylboronic acid
PVA	Polyvinyl Alcohol
MH	Minocycline Hydrochloride
Fe-Que NPs	Quercetin-Iron Nanoparticles
HP-PVA@MH/Fe-Que	ROS-responsive hydrogel
DPSC-EXO	Dental Pulp Stem Cell-derived Exosomes
MWHNPs@TAX	Taxifolin-Loaded Mesoporous Whitlockite Nanoparticles
GelMA	Methacrylated Gelatin
HUVECs	Human Umbilical Vein Endothelial Cells
*P.g*-LPS	*Porphyromonas gingivalis* Lipopolysaccharide
MZ	Metronidazole-loaded
MZ@PNM@GCP	Metronidazole-loaded targeted nanogels
BMSC-EVs	Extracellular Vesicles Derived from Bone Marrow Mesenchymal Stem Cells
CM-NPs	Cell Membrane-Coated Nanoparticles
